# Simple Clinical Prediction Rules for Identifying Significant Liver Fibrosis: Evaluation of Established Scores and Development of the Aspartate Aminotransferase-Thrombocytopenia-Albumin (ATA) Score

**DOI:** 10.3390/diagnostics15091119

**Published:** 2025-04-28

**Authors:** Puwitch Charoenchue, Jiraporn Khorana, Apichat Tantraworasin, Suwalee Pojchamarnwiputh, Wittanee Na Chiangmai, Amonlaya Amantakul, Taned Chitapanarux, Nakarin Inmutto

**Affiliations:** 1 Department of Radiology, Faculty of Medicine, Chiang Mai University, Chiang Mai 50200, Thailand; puwitch.c@cmu.ac.th (P.C.); suwalee.poj@cmu.ac.th (S.P.); wittanee.n@cmu.ac.th (W.N.C.); amonlaya.amantakul@cmu.ac.th (A.A.); 2 Department of Surgery, Faculty of Medicine, Chiang Mai University, Chiang Mai 50200, Thailand; apichat.t@cmu.ac.th; 3 Department of Biomedical Informatics and Clinical Epidemiology, Faculty of Medicine, Chiang Mai University, Chiang Mai 50200, Thailand; 4 Clinical Surgical Research Center, Faculty of Medicine, Chiang Mai University, Chiang Mai 50200, Thailand; 5 Department of Internal Medicine, Faculty of Medicine, Chiang Mai University, Chiang Mai 50200, Thailand; taned.chi@cmu.ac.th

**Keywords:** liver fibrosis, non-invasive tests, APRI, FIB-4, ATA score

## Abstract

**Background:** Existing non-invasive tests (NITs) for liver fibrosis offer moderate precision and accessibility but are often limited by complexity, reducing their practicality in routine clinical use. This study aimed to evaluate the diagnostic performance of current fibrosis assessment methods and develop a novel, simplified scoring system—the Aspartate Aminotransferase (AST)-Thrombocytopenia-Albumin (ATA) score—to enhance ease of use and clinical applicability. **Methods:** This study examined past cases of patients with chronic liver disease (CLD) by using magnetic resonance elastography (MRE) to evaluate fibrosis stages. Serum biomarkers were collected, and common fibrosis scores were calculated. Logistic regression identified potential predictors of significant fibrosis, forming the ATA score. Diagnostic performance was assessed, and internal validation was conducted via bootstrap resampling. **Results:** Among 70 patients, 31.4% had significant fibrosis. Hepatitis B was the most common cause (60.0%), followed by hepatitis C (18.6%) and nonalcoholic fatty liver disease (NAFLD, 15.7%). The ATA score demonstrated an area under the receiver operating characteristic curve (AUROC) of 0.872, comparable to the AST-to-platelet ratio index (APRI; 0.858) and fibrosis-4 index (FIB-4; 0.847). The recommended cut-offs for identifying high-risk patients were ATA score ≥ 2 (specificity 95.8%, sensitivity 50.0%), APRI ≥ 0.50 (specificity 89.6%, sensitivity 68.2%), and FIB-4 ≥ 1.3 (specificity 58.3%, sensitivity 90.9%). Internal validation confirmed model robustness, with an optimism-corrected AUROC of 0.8551. **Conclusions:** The ATA score offers a straightforward and efficient method for detecting significant fibrosis, demonstrating comparable diagnostic capability to APRI and FIB-4, while being more user-friendly in clinical practice. A score of 0–1 indicates low risk, suitable for clinical follow-up, whereas a score of ≥2 suggests high risk, warranting further evaluation. Integrating the ATA score into clinical workflows can enhance early detection, optimize resource utilization, and improve patient care.

## 1. Introduction

Chronic liver disease (CLD) is a growing global health burden, with fibrosis progression leading to cirrhosis and hepatocellular carcinoma. Early detection is essential for timely intervention and improved outcomes. Fibrosis develops due to sustained hepatic injury, leading to excessive extracellular matrix (ECM) deposition and liver architectural distortion [1]. The severity of fibrosis varies and is classified using multiple staging systems [2,3,4,5], with clinically significant fibrosis (≥METAVIR F2 or ≥Ishak score 3) serving as a key threshold for initiating therapeutic intervention [6].

The METAVIR scoring system is a commonly used pathological classification for liver fibrosis. Liver biopsy has long been the reference standard for assessing fibrosis severity [7]. However, biopsy is invasive, associated with risks such as bleeding, infection, and sampling variability, and is unsuitable for repeated follow-up assessments [7,8,9]. Moreover, because biopsy samples only a small portion of the liver, fibrosis heterogeneity may lead to sampling errors [10,11].

With advances in medical imaging, magnetic resonance elastography (MRE) has emerged as a highly accurate, non-invasive method for fibrosis assessment [12,13,14,15,16,17,18,19]. Compared to biopsy, MRE evaluates a larger liver volume, provides reproducible stiffness measurements, and minimizes procedural risks [20,21]. However, despite its diagnostic advantages, MRE remains expensive, time intensive, and may not be widely available, particularly in primary care and resource-limited settings [22].

To address this limitation, non-invasive tests (NITs) have been increasingly utilized as screening tools for identifying patients at risk for significant fibrosis. Among imaging-based methods, transient elastography (TE) and shear-wave elastography (SWE) are promising alternatives for screening and fibrosis staging [23]. However, limitations in early fibrosis, operator dependency, and limited accessibility continue to be concerns [22].

Researchers have developed serum biomarkers to enhance the accuracy of non-invasive fibrosis prediction by evaluating biochemical changes related to fibrosis. These biomarkers are classified into two main categories:

1. Direct biomarkers reflect ECM turnover and fibrogenesis, including hyaluronic acid, type III procollagen peptide, and matrix metalloproteinases. Although highly specific, these biomarkers often require specialized laboratory tests that may not be widely available.

2. Indirect biomarkers assess liver function and damage using commonly available blood tests, making them easier to implement in routine clinical settings [24].

Among the most widely used indirect biomarkers are the aspartate aminotransferase-to-alanine aminotransferase (AST/ALT) ratio [25], the AST-to-platelet ratio index (APRI) [26], and the fibrosis-4 index (FIB-4) [27]. These simple scoring systems rely on basic laboratory parameters, making them cost-effective options, particularly in primary care, where more advanced tests such as ultrasound elastography or MRE may not be accessible [24,28,29]. While serum biomarkers are effective at detecting advanced fibrosis, they have limited ability to distinguish significant fibrosis [24,30]. Consequently, further advancements in non-invasive prediction models are required to enhance early detection and risk stratification [31,32].

This study aims to (1) evaluate the diagnostic performance of established serum biomarker-based fibrosis prediction models (AST/ALT ratio, APRI, FIB-4) in detecting significant fibrosis and (2) develop and internally validate a novel, straightforward fibrosis prediction score using MRE as the reference standard. By comparing existing models with the newly proposed score, this study seeks to enhance the practicality of screening patients at risk for significant fibrosis while optimizing resource allocation and judiciously utilizing advanced imaging modalities, such as ultrasound elastography or MRE.

## 2. Materials and Methods

### 2.1. Study Design and Population

**Figure 1:**
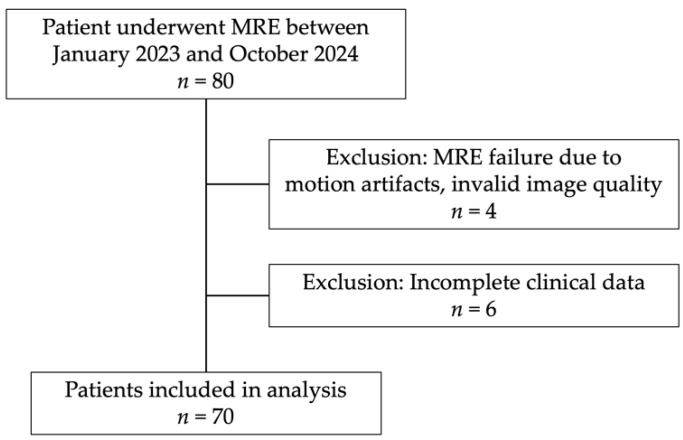
Flowchart of patient selection. A total of 80 patients underwent MRE. Four patients were excluded due to motion artifacts and invalid image quality, making liver stiffness measurement impossible, while six were excluded due to incomplete clinical data. The final analysis included 70 patients.

This retrospective study was conducted at a single tertiary university hospital as part of a cohort designed to evaluate liver fibrosis staging prospectively using MRE. This study included 80 adult patients (≥18 years old) with suspected CLD who underwent MRE between January 2023 and October 2024. Patients were referred for MRE due to abnormal liver function tests (LFTs) or clinical suspicion of cirrhosis. Of the initial cohort, 4 patients were excluded due to motion artifacts and invalid image quality, while 6 were excluded due to incomplete clinical data, leaving a final study population of 70 patients for analysis (Figure 1). All participants gave written informed consent, and the study protocol received approval from the Institutional Review Board (IRB).

### 2.2. Magnetic Resonance Elastography (MRE)

MRE examinations followed a standardized imaging protocol [33] to ensure consistency across all patients. Patients underwent a 4–6 h fasting period prior to imaging. During the scan, they were positioned supine, entering feet first into the scanner, and instructed to hold their breath at the end of exhalation. All MRE scans were performed using a 3.0-Tesla MRI system (GE Healthcare, Chicago, IL, USA). Mechanical vibrations were applied at 60 Hz using an external pneumatic driver, which was positioned over the liver at the right midclavicular line.

The imaging sequence utilized echo-planar imaging (EPI) to reduce motion artifacts, with the following acquisition parameters: a repetition time (TR) of 1000 ms, a flip angle of 90°, a field of view (FOV) of 420 mm with a matrix size of 96 × 96, and four axial slices (8 mm thick) with a 2 mm interslice gap, covering a broad liver area.

**Figure 2:**
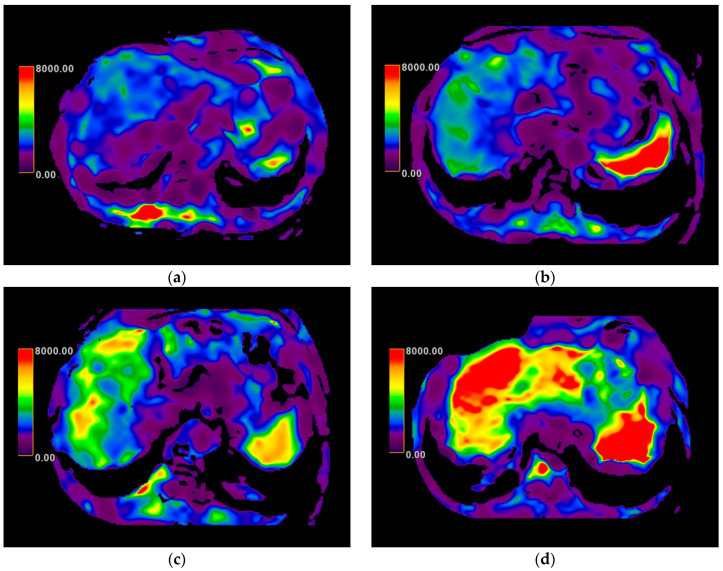
Magnetic resonance elastography (MRE) images showing liver stiffness in patients with different fibrosis stages: (**a**) normal (<2.5 kPa), (**b**) mild fibrosis (F1, 3.0–3.5 kPa), (**c**) significant fibrosis (F2, 3.5–4.0 kPa), and (**d**) cirrhosis (F4, >5.0 kPa). Stiffness values were measured using established cut-offs based on recommended guidelines. The color scale represents stiffness, with blue indicating lower and red higher values.

Figure 2 illustrates representative MRE-derived stiffness maps across fibrosis stages. MRE images were processed two weeks after acquisition by an independent investigator who was not provided with any clinical information to ensure unbiased results. Liver stiffness values were measured by placing regions of interest (ROIs) in four slices obtained from the liver stiffness map, ensuring a broad liver area was covered while avoiding major vessels and artifacts. The final liver stiffness was averaged across all four slices. Liver stiffness values were classified according to established cut-off thresholds for fibrosis severity [34,35,36,37,38]:

- <2.5 kPa → Normal liver stiffness.
- 2.5–3.0 kPa → Normal or potential inflammation.
- 3.0–3.5 kPa → Early fibrosis (Stage 1–2).
- 3.5–5.0 kPa → Significant fibrosis (F2–F3).
- >5.0 kPa → Cirrhosis (F4).

### 2.3. Clinical Variables

Clinical data were obtained within one month of the MRE examination. Collected variables included age, sex, body mass index (BMI), platelet count, ALT, AST, alkaline phosphatase (ALP), total bilirubin, and cholesterol levels. Fibrosis assessment scores, including the AST/ALT ratio, the APRI, and the FIB-4, were calculated. Liver stiffness measurements from MRE (kPa) were categorized according to fibrosis stages.

### 2.4. Statistical Analysis

Comparative analysis was performed between patients with normal or mild fibrosis (F0–F1) and those with significant fibrosis. The diagnostic performance of the AST/ALT ratio, APRI, FIB-4, and the newly developed fibrosis prediction score was assessed using area under the receiver operating characteristic curve (AUROC) analysis. Sensitivity, specificity, positive predictive value (PPV), negative predictive value (NPV), and positive likelihood ratio (LR+) were calculated. Univariate and multivariate logistic regression analyses were performed to identify independent predictors of significant fibrosis. A new fibrosis prediction score was developed using selected predictors, with coefficients derived from regression models, and various cut-off values were tested for optimal diagnostic accuracy. Calibration plots were generated to assess model fit, and internal validation was conducted using bootstrap resampling. Statistical analyses were conducted using STATA software (version 18.5, StataCorp LLC, College Station, TX, USA), with statistical significance defined as *p* < 0.05.

### 2.5. Sample Size Calculation

We estimated the sample size for developing a clinical prediction model using the “pmsampsize” method in STATA, as described by Riley et al. [39]. The calculation incorporated an expected Nagelkerke’s R^2^ of 0.3, three predictor variables, and an outcome prevalence of 0.314 (based on the study population). Based on Criteria 1 and 2, which aim to minimize overfitting and maintain stable model performance, the estimated sample size ranged between 70 and 74 patients, ensuring an events-per-predictor (EPP) ratio between 7.33 and 7.75. Given that our study included 70 patients, the sample size meets the minimum threshold required for prediction model development.

While a larger sample size (331 patients) was suggested under Criterion 3 for precise population risk estimation, our study adhered to Criteria 1 and 2, which provide a balance between feasibility and model stability. To mitigate potential overfitting, bootstrap validation with 1234 replications was performed to correct optimism in the model’s performance.

### 2.6. Ethics Approval

This study was conducted in accordance with the Declaration of Helsinki and received approval from the IRB (Study Code: RAD-2564-08543, EC certificate No. 353/2022, approval date: 17 October 2022).

## 3. Results

### 3.1. Patient Characteristics

The analysis included a total of 70 patients, of which 22 (31.4%) were identified as having significant fibrosis. The median age was 54.5 years (range: 23–74), and 58.6% were male. The leading etiology of chronic liver disease was hepatitis B (60.0%), followed by hepatitis C (18.6%), nonalcoholic fatty liver disease (NAFLD) (15.7%), alcoholic liver disease (11.4%), autoimmune hepatitis (1.4%), and iron overload (5.7%). Baseline characteristics stratified by fibrosis severity are presented in Table 1.

Patients with significant fibrosis were generally older (58.5 vs. 52.5 years) and had a higher proportion of males (72.73% vs. 52.08%) compared to those with normal or mild fibrosis. Statistically significant differences were observed in platelet count (138.5 vs. 215.5 × 10^3^/mm^3^), serum albumin (4.35 vs. 4.5 g/dL), AST (38 vs. 24 U/L), ALT (32.5 vs. 21.5 U/L), and cholesterol (146 vs. 197 mg/dL), with the significant fibrosis group exhibiting lower platelet count, lower albumin levels, and lower cholesterol levels, while having higher AST and ALT levels.

**Figure 3:**
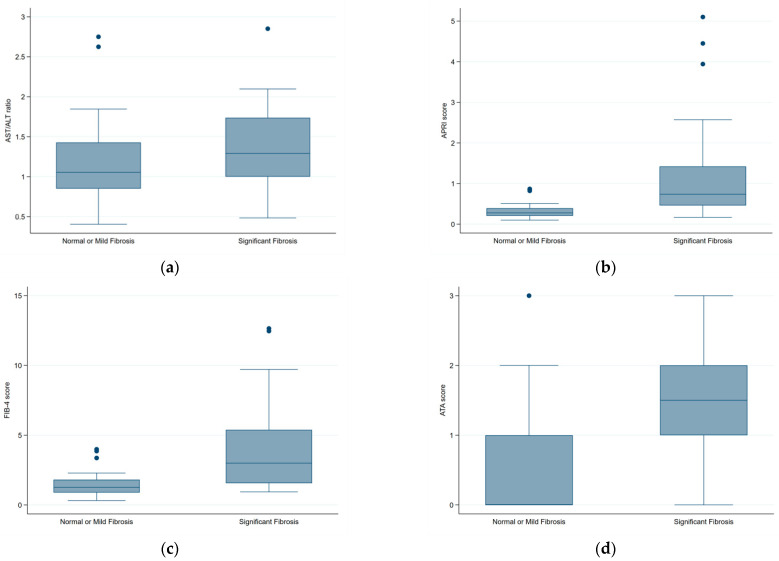
Box plots illustrating the distribution of (**a**) AST/ALT ratio, (**b**) APRI score, (**c**) FIB-4 score, and (**d**) ATA score across fibrosis severity groups (normal or mild fibrosis vs. significant fibrosis). The Mann–Whitney U test showed significant differences for APRI, FIB-4, and ATA scores (*p* < 0.001) but not for the AST/ALT ratio (*p* = 0.08). Each box plot illustrates the median, interquartile range (IQR), and outliers for each variable.

Similarly, APRI (0.74 vs. 0.28) and FIB-4 (2.99 vs. 1.26) scores were notably higher in patients with significant fibrosis, while the AST/ALT ratio (1.29 vs. 1.05) showed a slight increase but did not reach statistical significance. Box plots illustrating the distribution of these fibrosis-scoring models across groups are shown in Figure 3.

### 3.2. Development of a Diagnostic Model for Significant Fibrosis

To identify clinical variables with diagnostic value in predicting significant fibrosis, we performed univariable and multivariable logistic regression analyses. Both continuous and binary forms of laboratory parameters were evaluated during model development. Specifically, we compared the discriminative performance of continuous predictors versus dichotomized variables using clinically relevant cut-off points. We found that using simple thresholds—closely aligned with standard reference ranges—preserved diagnostic performance while improving interpretability and ease of use. This approach enabled us to avoid a complex linear model and instead construct a practical, point-based scoring system.

Based on statistical significance and clinical relevance, we selected three predictors for the final model:

- Thrombocytopenia (platelet count < 150 × 10^3^/mm^3^).
- AST ≥ 30 U/L.
- Hypoalbuminemia (serum albumin ≤ 3.5 g/dL).

These three variables were then incorporated into a new diagnostic model, termed the AST-thrombocytopenia-albumin (ATA) Score. As shown in Table 2, these predictors demonstrated strong diagnostic performance, with odds ratios ranging from 6.84 to 9.28 and regression coefficients between 1.92 and 2.23. Thrombocytopenia and AST ≥ 30 U/L were statistically significant (*p* = 0.006 and *p* = 0.001, respectively), while hypoalbuminemia (*p* = 0.190) did not reach statistical significance but was retained based on its known clinical relevance in chronic liver disease.

To enhance ease of use, we developed a simplified point-based scoring system by assigning 1 point to each predictor. This decision was supported by the similarity of their regression coefficients, suggesting comparable contributions to the risk of significant fibrosis. Equal weighting facilitates bedside application and avoids complex calculations, making the ATA score (range: 0–3) a practical, non-invasive tool for identifying at-risk patients.

### 3.3. Diagnostic Performance of the Prediction Scoring System

**Figure 4:**
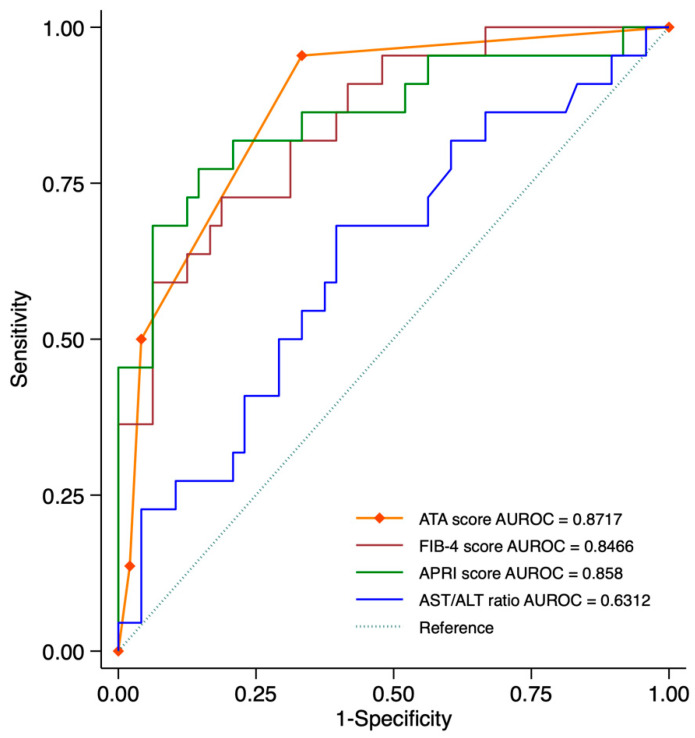
Receiver operating characteristic (ROC) curves comparing the diagnostic performance of the ATA score, APRI score, FIB-4 score, and AST/ALT ratio for identifying significant fibrosis (≥F2, MRE ≥ 3.5 kPa). The area under the curve (AUROC) for each scoring system is displayed in the legend: ATA score (AUROC = 0.8717), APRI score (AUROC = 0.858), FIB-4 score (AUROC = 0.8466), and AST/ALT ratio (AUROC = 0.6312). The ATA score demonstrates the highest AUROC among the four models, suggesting superior diagnostic performance.

The ATA score demonstrated the highest AUROC (0.8717) among the four models. However, the AUROC values of ATA, APRI (0.858), and FIB-4 (0.8466) were not significantly different, suggesting comparable diagnostic performance in identifying significant fibrosis. In contrast, the AST/ALT ratio had a significantly lower AUROC (0.6312, *p* < 0.05) compared to the other models, indicating inferior diagnostic utility (Figure 4).

To further assess the ATA score’s performance, we compared its AUROC against each individual continuous laboratory parameter included in the score, serum AST, platelet count, albumin level, and ALT, which is commonly included in liver function panels. The ATA score exhibited the highest diagnostic accuracy, significantly outperforming each individual variable (*p* = 0.0072) (Supplementary Figure S1).

To further evaluate diagnostic accuracy, we assessed various cut-off values for each scoring system, as summarized in Table 3. The ATA score (cut-off ≥ 2) achieved a high specificity (95.8%) and accuracy (81.4%), while maintaining a positive likelihood ratio (LR+) of 12.00, making it a strong predictive tool. Similarly, the APRI score (cut-off 0.50) showed high accuracy (82.9%) and an AUROC of 0.79. For FIB-4, a cut-off of 1.3 demonstrated moderate performance with an AUROC of 0.73, a sensitivity of 90.9%, and a specificity of 54.2%, providing a balance between ruling out and detecting significant fibrosis. Additionally, the FIB-4 cut-off at 1.4 yielded a slightly improved AUROC of 0.75, though with lower sensitivity (90.9%) and higher specificity (58.3%). In contrast, the AST/ALT ratio had the lowest AUROC (0.6312), further reinforcing its limited diagnostic value compared to the other models.

These findings indicate that the ATA score provides a straightforward and effective method for predicting significant fibrosis, demonstrating performance comparable to APRI and FIB-4, while surpassing the AST/ALT ratio.

### 3.4. Calibration and Internal Validation

**Figure 5:**
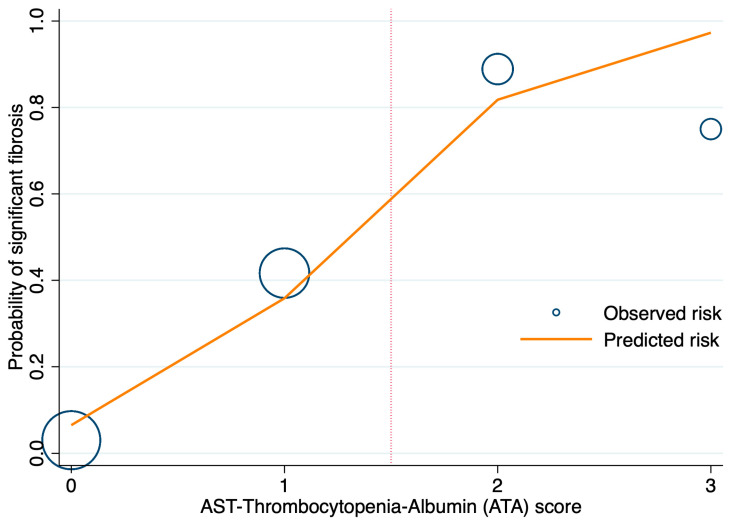
Calibration plot for the AST-thrombocytopenia-albumin (ATA) score in predicting significant fibrosis. The orange line represents predicted probabilities, and blue circles indicate observed probabilities for each ATA score category, with circle sizes proportional to the number of patients. ATA scores of 0–1 are classified as low risk, while scores of 2–3 are classified as high risk for significant fibrosis. The vertical dotted line visually separates the low-risk and high-risk categories. The plot demonstrates good calibration between observed and predicted probabilities, supporting the reliability of the ATA score.

Calibration analysis demonstrated strong agreement between observed and predicted probabilities of significant fibrosis across ATA score categories (Figure 5). ATA scores of 0–1 corresponded to a low probability of significant fibrosis, whereas scores of 2–3 suggested a higher risk, supporting the practical use of ATA ≥ 2 as a threshold for high-risk classification.

To ensure internal validity, bootstrap validation with 1234 replications was performed. The apparent AUROC was 0.8706 (95% CI: 0.7885–0.9435), while the bias-corrected AUROC was 0.8551 (95% CI: 0.8139–0.9180), with an optimism value of 0.0155. These results indicate minimal overfitting and strong generalizability of the ATA score in identifying significant fibrosis.

## 4. Discussion

### 4.1. Non-Invasive Tools for Liver Fibrosis Assessment

CLD progresses to fibrosis at varying degrees, necessitating efficient diagnostic tools for the early detection of significant fibrosis. Although liver biopsy is the definitive diagnostic method, it is invasive, expensive, and carries risks such as bleeding and sampling variability. As a result, NITs, including imaging elastography and serum biomarkers, have gained clinical relevance as alternative methods for fibrosis assessment [40].

Imaging-based techniques such as MRE, TE, and SWE provide reliable fibrosis staging but require specialized equipment and trained operators, limiting their accessibility in primary care settings [41]. Serum biomarkers offer a more practical alternative, with direct biomarkers (e.g., hyaluronic acid, procollagen peptides) better reflecting extracellular matrix remodeling, though they remain costly and less available. In contrast, indirect biomarkers serve as cost-effective tools for fibrosis screening, forming the basis of various CPRs.

### 4.2. Diagnostic Performance of Existing Clinical Prediction Scores

#### 4.2.1. AST/ALT Ratio

The AST/ALT ratio was first introduced in 1957 for diagnosing viral hepatitis and has since been explored as a marker for liver fibrosis [42]. Subsequent studies have suggested that an AST/ALT ratio ≥ 1 is associated with advanced fibrosis, while a ratio ≥ 2 strongly suggests cirrhosis [25,43,44]. However, its clinical utility is limited due to poor specificity, as various hepatic and non-hepatic conditions can influence the ratio. The AST/ALT ratio is also useful in differentiating nonalcoholic steatohepatitis (NASH) from alcoholic liver disease, with a ratio ≥ 2 favoring alcoholic liver disease, while a ratio < 1 is more indicative of NASH [45]. However, its predictive accuracy varies across different liver diseases, making it insufficient as a standalone marker for fibrosis staging [44].

#### 4.2.2. AST-to-Platelet Ratio Index (APRI)

The APRI score, calculated as [AST (IU/L)/AST upper limit of normal (ULN)]/platelet count (×10^9^/L), was initially developed for chronic hepatitis C (CHC) and has been widely used to assess significant fibrosis and cirrhosis [26]. Across different causes of liver fibrosis, the APRI identified significant fibrosis with an AUROC of 0.76, indicating moderate diagnostic performance [46]. However, its diagnostic performance depends significantly on the selected threshold.

Commonly used cut-off values for APRI vary based on fibrosis severity. A threshold of 0.5 is frequently used to identify significant fibrosis, with a sensitivity of 0.74 to 0.82 and a specificity of 0.49 to 0.75 [26,46,47].

A meta-analysis by Lin et al. (2010) reported that an APRI threshold of 0.7 provided the optimal balance between sensitivity (77%) and specificity (72%) for detecting significant fibrosis in patients with chronic hepatitis C [47]. A lower cut-off of 0.5 yielded higher sensitivity (74%) but lower specificity (49%), making it useful for ruling out fibrosis. Conversely, a higher cut-off of 1.5 demonstrated a specificity of 93% but reduced sensitivity to 37%, making it more useful for confirming significant fibrosis [47].

In our study, APRI revealed an AUROC of 0.86 for significant fibrosis, comparable to previous reports. The optimal cut-off in our cohort was 0.50, achieving a sensitivity of 68.2% and specificity of 89.6%, with an accuracy of 82.9%. This suggests that APRI ≥ 0.50 serves as a reasonable threshold for identifying patients at risk of significant fibrosis who may require further evaluation, such as ultrasound elastography or MRE.

Furthermore, APRI values ≥ 0.80 improved specificity (93.8%) but reduced sensitivity (45.5%), while a higher cut-off of 1.0 reached 100% specificity, confirming fibrosis with high confidence but at the cost of reduced sensitivity. These findings align with previous studies, reinforcing the utility of APRI as an accessible and practical tool for fibrosis screening, with appropriate threshold selection based on clinical application.

#### 4.2.3. FIB-4 Index

The FIB-4 index, calculated as [age (years) × AST (U/L)]/[platelet count (×10^9^/L) × √ALT (U/L)], was originally developed for HIV/HCV co-infected patients but is now widely used for NAFLD and other liver diseases. Several studies have attempted to establish appropriate FIB-4 cut-off values for fibrosis staging. While the original FIB-4 study by Sterling et al. (2006) suggested 1.45 and 3.25 as cut-offs for distinguishing significant fibrosis and advanced fibrosis in HIV/HCV co-infected patients, these thresholds may not be universally applicable across different liver diseases [27].

For NAFLD, a cut-off of 1.3 has gained increasing support for excluding advanced fibrosis. The Hepatology Communications (2019) study found that FIB-4 < 1.3 corresponded to a low probability (~12.5%) of advanced fibrosis (VCTE ≥ 8 kPa), making it an effective triage tool. Moreover, this threshold prioritizes patients for additional workup, reducing unnecessary referrals and optimizing the use of elastography [48].

However, applying a single cut-off across different patient populations presents challenges. The performance of FIB-4 varies with age, and studies have shown that age-adjusted thresholds (e.g., 1.3 for patients < 65 years and 2.0 for older individuals) improve specificity [48]. Additionally, conditions like diabetes and obesity may influence FIB-4 accuracy, requiring careful clinical interpretation [48].

In our study, we used a cut-off of 1.3 for significant fibrosis, aligning with the recent literature. Patients with FIB-4 ≥ 1.3 had a higher probability of significant fibrosis and should undergo further assessment with MRE or ultrasound elastography to confirm disease severity. This approach ensures early detection while minimizing unnecessary testing in low-risk individuals.

In NASH patients, FIB-4 achieved the highest AUROC (0.86) for advanced fibrosis, outperforming other CPRs [49]. A meta-analysis reported AUROCs of 0.74 and 0.87 for significant fibrosis and cirrhosis, respectively [50]. Given their accessibility, APRI and FIB-4 are recommended by the WHO for fibrosis assessment but should be used alongside other diagnostic tools [51].

### 4.3. The ATA Score Compared to Existing Scores

The ATA score was developed using three key laboratory parameters that are closely associated with liver fibrosis: AST, platelet count, and serum albumin. These biomarkers reflect different but complementary pathophysiological aspects of fibrosis progression. Platelets play a crucial role in liver fibrosis assessment, as a decrease in platelet count is commonly observed in patients with advanced fibrosis and cirrhosis.

- Thrombocytopenia in liver fibrosis is primarily attributed to hypersplenism due to portal hypertension, as well as reduced thrombopoietin production by the liver [52]. Studies have demonstrated a strong inverse correlation between platelet count and fibrosis severity, making it a reliable surrogate marker for non-invasive fibrosis staging [53,54].
- AST, an enzyme released by the liver during hepatocyte injury, is commonly used as a biomarker for monitoring liver fibrosis progression. AST elevation correlates with ongoing inflammation and hepatocyte turnover, making it a sensitive indicator of significant fibrosis. Several studies have identified AST as an independent predictor of fibrosis, with persistently high AST levels being associated with faster fibrosis progression [55,56,57]. Although ALT is considered more specific to hepatocellular injury [58], we selected AST for inclusion based on both statistical performance and its closer association with hepatic fibrosis. AST is present in both the cytoplasm and mitochondria of hepatocytes, and mitochondrial AST release has been linked to chronic hepatic injury and fibrogenesis. Prior studies have demonstrated that elevated AST levels—and particularly a higher AST/ALT ratio—are predictive of advanced fibrosis and cirrhosis in patients with chronic liver disease [25,59,60]. In our cohort, AST showed stronger predictive value for significant fibrosis than ALT, supporting its inclusion in the final model. This selection also aligns with our goal of optimizing diagnostic performance while minimizing redundancy in closely related biochemical markers.
- Serum albumin, a liver-synthesized protein, is an essential marker of liver function. A decline in albumin levels signifies hepatic dysfunction and is frequently detected in patients with advanced fibrosis and cirrhosis [61]. Studies have shown that lower albumin levels are associated with severe fibrosis and liver-related complications. Additionally, albuminuria has been linked to liver fibrosis severity, suggesting that hypoalbuminemia may indicate systemic consequences of chronic liver disease [62,63,64].

Our findings demonstrated that APRI and FIB-4 were acceptable in predicting significant fibrosis, with AUROCs comparable to prior studies [26,27]. These CPRs have been validated in external cohorts [47,65,66,67]. However, one limitation of these models is their computational complexity, making them less convenient for point-of-care assessment. The newly developed ATA score addresses these limitations by utilizing only three predictors, making it simpler to apply in routine clinical practice.

### 4.4. Sample Size Considerations

Our study included 70 patients, which aligns with the minimum recommended sample size based on Criteria 1 and 2 from the “pmsampsize” method [39], which prioritizes minimizing overfitting and ensuring stable model performance [39]. Although larger sample sizes are generally preferred for clinical prediction models, recent studies have demonstrated that models with a lower events-per-predictor (EPP) ratio can still maintain acceptable predictive performance, especially when appropriate validation techniques are applied [68,69].

To ensure model reliability despite the limited sample size, bootstrap validation with 1234 replications was performed, yielding an optimism-corrected AUROC of 0.8551, which supports the model’s predictive accuracy. Bootstrap resampling has been recognized as an effective method for reducing overfitting and improving model stability, even in datasets with a lower EPP ratio [70].

While a larger external validation cohort is warranted to further confirm the model’s broader applicability, our findings demonstrate that small, well-validated prediction models can provide clinically useful insights for identifying significant fibrosis.

### 4.5. Strengths and Limitations

Our study used MRE as the reference standard, aligning fibrosis staging with the METAVIR classification. While MRE provides a clinically useful alternative to biopsy, it is not without limitations. Although MRE has been validated in numerous studies with high accuracy for detecting liver fibrosis, it may not be as conclusive as histopathological assessment from liver biopsy, which continues to be the gold standard for fibrosis staging [71]. Additionally, MRE is susceptible to motion artifacts and technical variability, which may affect fibrosis classification in certain cases [34]. The ATA score demonstrated comparable discrimination power to APRI and FIB-4, suggesting its utility as an accessible alternative.

Despite these strengths, several limitations must be acknowledged. While meeting the minimum threshold for model development, the sample remains below the ideal size for precise risk estimation. Furthermore, our study was conducted in a tertiary referral center, where the prevalence and severity of liver fibrosis may be higher than in primary care settings, potentially affecting the generalizability of the findings. Another potential limitation is the heterogeneity of our cohort, which included patients with various underlying liver diseases (e.g., viral hepatitis, NAFLD, and autoimmune liver disease). This diversity may have influenced the cut-off levels for fibrosis prediction. In particular, alcoholic liver disease—a major global contributor to liver fibrosis—was underrepresented in our study population. As a result, the ATA score’s performance may not be fully generalizable to populations with a higher prevalence of alcohol-related liver injury. This underrepresentation should be considered when interpreting the findings, and future validation efforts should prioritize cohorts that reflect a broader etiologic distribution. However, this heterogeneity may also be a strength, as it suggests that the ATA score can be applied across different etiologies, enhancing its real-world applicability.

Another key limitation is the absence of an external validation cohort. While internal validation was performed using bootstrap resampling, these findings have not yet been tested in an independent population. As a result, the generalizability of the ATA score remains uncertain.

Future studies should validate the ATA score externally across diverse healthcare settings and patient populations, including primary care and non-specialist environments, to confirm its generalizability. Additionally, research should explore refinements of the score by incorporating other relevant biomarkers or applying it to more varied clinical contexts.

## 5. Conclusions

The ATA score is a simple, effective tool for identifying significant fibrosis, matching the diagnostic performance of APRI and FIB-4 while being easier to use in clinical settings. Based on our findings, an APRI cut-off of ≥0.50, a FIB-4 cut-off of ≥1.3, and an ATA score cut-off of ≥2 are recommended for identifying high-risk patients who should undergo further evaluation with ultrasound elastography or magnetic resonance elastography to confirm fibrosis and guide management. Conversely, lower values may indicate a lower risk, suitable for clinical follow-up without immediate advanced imaging. Integrating these non-invasive scoring systems into routine workflows can optimize fibrosis screening, improve early detection, and reduce unnecessary tests, ultimately enhancing patient care and resource allocation.

## Figures and Tables

**Table 1:** Baseline characteristics of patients stratified by fibrosis severity into normal or mild fibrosis and significant fibrosis.

Characteristics	Normal or Mild Fibrosis *n* = 48 (68.6%)	Significant Fibrosis *n* = 22 (31.4%)	*p*-Value
Age (years), median (range)	52.5 (23–74)	58.5 (40–70)	0.15
Sex (male, %)	25 (52.08%)	16 (72.73%)	0.12
Body Mass Index, median (IQR)	24.69 (21.96–26.73)	23.79 (20.90–28.39)	0.89
Platelet count (×10^3^/mm^3^), median (IQR)	215.5 (170.5–271.5)	138.5 (89.0–191.0)	<0.001
Total Protein (g/dL), median (IQR)	7.75 (7.5–8.0)	7.80 (7.7–8.1)	0.34
Serum Albumin (g/dL), median (IQR)	4.5 (4.4–4.6)	4.35 (3.9–4.4)	<0.001
AST (U/L), median (IQR)	24 (21–28)	38 (30–84)	<0.001
ALT (U/L), median (IQR)	21.5 (15.5–30.5)	32.5 (22–58)	0.007
Cholesterol (mg/dL), median (IQR)	197 (164.5–219.5)	146 (122–169)	<0.001
AST/ALT ratio, median (IQR)	1.05 (0.85–1.43)	1.29 (1.00–1.74)	0.08
APRI score, median (IQR)	0.28 (0.20–0.39)	0.74 (0.46–1.42)	<0.001
FIB-4 score, median (IQR)	1.26 (0.89–1.81)	2.99 (1.56–5.37)	<0.001

**Table 2:** Regression coefficient, diagnostics odds ratio (dOR), and 95% CI of selected diagnostic parameters derived from logistic regression.

Predictor	Coefficient	Odds Ratio	95% CI for OR	*p*-Value
Thrombocytopenia	1.96	7.10	1.74–28.99	0.006
AST ≥ 30 U/L	2.23	9.28	2.42–35.62	0.001
Albumin ≤ 3.5 g/dL	1.92	6.84	0.39–121.34	0.190

**Table 3:** Diagnostic performance of FIB-4 score, APRI score, AST/ALT Ratio, and ATA score for identifying significant fibrosis.

Score	Cut-Off	Sensitivity (%)	Specificity (%)	PPV (%)	NPV (%)	LR (+)	AUROC (95% CI)	Accuracy (%)
AST/ALT	1.0	77.3	39.6	37.0	79.2	1.28	0.58 (0.47–0.70)	51.4
	1.2	59.1	60.4	40.6	76.3	1.49	0.60 (0.47–0.72)	60.0
	1.25	54.5	66.7	42.9	76.2	1.64	0.61 (0.48–0.73)	62.9
APRI	0.245	95.5	41.7	42.9	95.2	1.64	0.69 (0.60–0.77)	58.6
	0.39	81.8	75.0	60.0	90.0	3.27	0.78 (0.68–0.89)	77.1
	0.50	68.2	89.6	75.0	86.0	6.55	0.79 (0.68–0.90)	82.9
	0.70	50.0	93.8	78.6	80.4	8.00	0.72 (0.61–0.83)	80.0
	0.80	45.5	93.8	76.9	78.9	7.27	0.70 (0.58–0.81)	78.6
	1.0	45.5	100.0	100.0	80.0	-	0.73 (0.62–0.83)	82.9
FIB-4	1.0	95.5	35.4	40.4	94.4	1.48	0.65 (0.57–0.74)	54.3
	1.2	95.5	43.8	43.8	95.5	1.70	0.70 (0.61–0.78)	60.0
	1.3	90.9	54.2	47.6	92.9	1.98	0.73 (0.63–0.82)	65.7
	1.4	90.9	58.3	50.0	93.3	2.18	0.75 (0.65–0.84)	68.6
	1.5	81.8	64.6	51.4	88.6	2.31	0.73 (0.62–0.84)	70.0
	2.0	72.7	81.3	64.0	86.7	3.88	0.77 (0.66–0.88)	78.6
ATA	1	95.5	66.7	56.8	97.0	2.86	0.81 (0.73–0.89)	75.7
	2–3	50.0	95.8	84.6	80.8	12.00	0.73 (0.62–0.84)	81.4

## Data Availability

Data supporting the findings of this study are not publicly available due to Thailand’s Personal Data Protection Act (PDPA) but may be provided upon an official request and approval.

## Supplementary Materials

The following supporting information can be downloaded at: https://www.mdpi.com/article/10.3390/diagnostics15091119/s1, Figure S1: ROC curves compare the diagnostic performance of the composite ATA score versus individual laboratory parameters (AST, ALT, albumin, and platelet count) to identify significant liver fibrosis. The ATA score achieved the highest AUROC (0.872), significantly higher than AST (0.816), albumin (0.760), platelet count (0.787), and ALT (0.699), with a global comparison *p*-value of 0.0072.
