# Supplementary material for: Simple Clinical Prediction Rules for Identifying Significant Liver Fibrosis: Evaluation of Established Scores and Development of the Aspartate Aminotransferase-Thrombocytopenia-Albumin (ATA) Score
Source: Diagnostics (Basel). 2025 Apr 28;15(9):1119. doi: 10.3390/diagnostics15091119 (PMC12071440; doi:10.3390/diagnostics15091119)
Supplement: Supplementary file 1 [file diagnostics-15-01119-s001.zip › diagnostics-3569798-Supplementary.pdf]

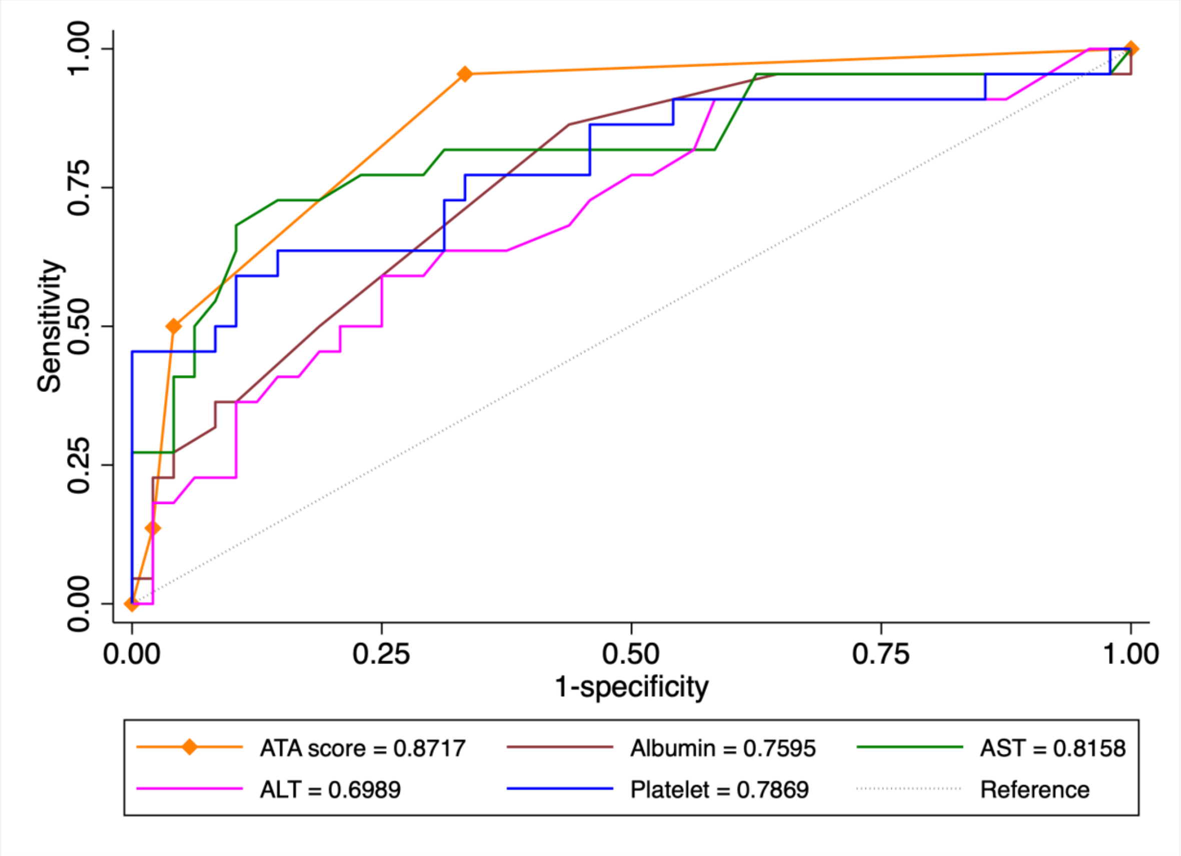

Supplementary Figure S1. ROC curves compare the diagnostic performance of the composite ATA Score versus individual laboratory parameters (AST, ALT, albumin, and platelet count) to identify significant liver fibrosis. The ATA Score achieved the highest AUROC (0.872), significantly higher than AST (0.816), albumin (0.760), platelet count (0.787), and ALT (0.699), with a global comparison *p*-value of 0.0072.
